# Adjuvant hypofractionated radiotherapy with simultaneous integrated boost after breast-conserving surgery: results of a prospective trial

**DOI:** 10.1007/s00066-020-01689-7

**Published:** 2020-10-01

**Authors:** David Krug, René Baumann, Katja Krockenberger, Reinhard Vonthein, Andreas Schreiber, Alexander Boicev, Florian Würschmidt, Evelyn Weinstrauch, Kirsten Eilf, Peter Andreas, Ulrike Höller, Stefan Dinges, Karen Piefel, Jörg Zimmer, Kathrin Dellas, Jürgen Dunst

**Affiliations:** 1 Klinik für Strahlentherapie, Universitätsklinikum Schleswig-Holstein, Arnold-Heller-Str. 3, 24105 Kiel, Germany; 2 Klinik für Radio-Onkologie, St. Marien-Krankenhaus Siegen, Siegen, Germany; 3 ZKS Lübeck, Universität zu Lübeck, Lübeck, Germany; 4 Institut für Medizinische Biometrie und Statistik, Universität zu Lübeck, Lübeck, Germany; 5 Praxis für Strahlentherapie Dr. med. Andreas Schreiber, Dresden, Germany; 6 Klinik für Strahlentherapie und Radioonkologie, Heinrich-Braun-Klinikum Zwickau, Zwickau, Germany; 7 Radiologische Allianz, Hamburg, Germany; 8 Praxis für Radioonkologie, Johanniter-Zentren für Medizinische Versorgung Stendal, Stendal, Germany; 9 Praxis für Strahlentherapie, Kiel, Germany; 10 Abteilung für Strahlentherapie, Krankenhaus Buchholz, Buchholz in der Nordheide, Germany; 11 MVZ Charité Vivantes, Berlin, Germany; 12 Klinik für Strahlentherapie & Radioonkologie, Lüneburg, Germany; 13 Strahlenzentrum Hamburg MVZ, Hamburg, Germany; 14 AMEDOM GmbH, Lübeck, Germany; 15 Fachbereich Strahlentherapie, Ambulanzzentrum des UKE, Hamburg, Germany

**Keywords:** Radiation dermatitis, Acute toxicity, Intensity modulated radiotherapy, Treatment compliance, Adverse events

## Abstract

**Purpose:**

We report results of a multicenter prospective single-arm phase II trial (ARO-2013-04, NCT01948726) of moderately accelerated hypofractionated radiotherapy with a simultaneous integrated boost (SIB) in patients with breast cancer receiving adjuvant radiotherapy after breast-conserving surgery.

**Methods:**

The eligibility criteria included unifocal breast cancer with an indication for adjuvant radiotherapy to the whole breast and boost radiotherapy to the tumor bed. The whole breast received a dose of 40 Gy and the tumor bed a total dose of 48 Gy in 16 fractions of 2.5 and 3 Gy, respectively. Radiotherapy could be given either as 3D conformal RT (3D-CRT) or as intensity-modulated radiotherapy (IMRT). The study was designed as a prospective single-arm trial to evaluate the acute toxicity of the treatment regimen. The study hypothesis was that the frequency of acute skin reaction grade ≥2 would be 20% or less.

**Results:**

From November 2013 through July 2014, 149 patients were recruited from 12 participating centers. Six patients were excluded, leaving 143 patients for analysis. Eighty-four patients (58.7%) were treated with 3D-CRT and 59 (41.3%) with IMRT. Adherence to the treatment protocol was high. The rate of grade ≥2 skin toxicity was 14.7% (95% confidence interval 9.8–21.4%). The most frequent grade 3 toxicity (11%) was hot flashes.

**Conclusion:**

This study demonstrated low toxicity of and high treatment adherence to hypofractionated adjuvant radiotherapy with SIB in a multicenter prospective trial, although the primary hypothesis was not met.

## Introduction

Moderate hypofractionation has become standard of care for adjuvant whole-breast radiotherapy. Several randomized controlled trials and a Cochrane meta-analysis have demonstrated equal results in terms of local control and late toxicity as compared to conventional fractionation [1]. In the relevant randomized controlled trials, the boost to the tumor bed, if administered, was given as sequential boost after irradiation of the whole breast, increasing the treatment time by 1 to 1.5 weeks [2–4].

Recently, the simultaneous integrated boost (SIB) technique has been introduced in combination with conventionally fractionated whole-breast irradiation (WBI). The SIB improves dose homogeneity, reduces overdosage in the breast outside the boost volume, and improves the biologically effective dose–volume histogram [5–7]. Results from a prospective non-randomized trial using conventionally fractionated whole-breast radiotherapy with a SIB to the tumor bed demonstrated excellent local control rates [8]. Both conventionally fractionated radiotherapy with SIB and hypofractionated radiotherapy with a sequential boost are currently accepted as standard regimens for adjuvant radiotherapy after breast-conserving surgery and reduce the overall treatment time by about 1.5 to 2 weeks as compared to conventionally fractionated whole-breast radiotherapy with a sequential boost [9, 10]. The combination of hypofractionated WBI with a SIB can further shorten the treatment time to about 3 weeks.

First clinical results of hypofractionated whole-breast radiotherapy with SIB [11–15] suggest that incorporating SIB into hypofractionated WBI is feasible, although patient numbers are small and follow up is limited.

We previously conducted a prospective trial (ARO-2010-01) investigating the feasibility of hypofractionation with SIB [13]. On the basis of these results, we have conducted a second study to further investigate acute toxicity in preparation of a randomized controlled phase III trial (HYPOSIB; NCT02474641).

## Materials and methods

ARO-2013-04 was a multicenter prospective single-arm trial investigating the toxicity profile of moderately accelerated hypofractionated radiotherapy (16 fractions) with a SIB in patients receiving adjuvant radiotherapy after breast-conserving surgery.

### Eligibility criteria

The eligibility criteria included women aged 18 years or older with histologically confirmed unifocal breast cancer who had undergone breast-conserving surgery with clear margins. Patients were scheduled to receive adjuvant radiotherapy to the whole breast (without regional lymph nodes) including boost radiotherapy to the tumor bed. Prior chemotherapy (either as adjuvant or neoadjuvant treatment) was allowed. Endocrine therapy was also allowed during radiotherapy. Main exclusion criteria were mastectomy, no indication for a boost, no clear identification of tumor bed on planning computed tomography (CT), extensive seroma after surgery necessitating excessive boost volume, indication for irradiation of regional nodes, previous chest radiotherapy, and relevant comorbidity limiting the administration of protocol-specific radiotherapy.

Pretreatment evaluation included a complete history and physical examination. Staging procedures were performed according to national and international guidelines for diagnosis and treatment of breast cancer [16, 17]. The tumor bed had to be clearly identifiable (i.e., through seroma or tumor bed clips) on planning CT. The use of tumor bed clips was not mandatory. Informed consent was obtained from all patients.

### Radiation therapy

The treatment protocol was identical to the preceding feasibility study [13]. Briefly, the whole breast received a dose of 40 Gy in 16 fractions of 2.5 Gy. A SIB with an additional dose of 0.5 Gy per fraction was administered to the tumor bed, resulting in a total dose of 48 Gy in 16 fractions to the boost planning target volume (PTV). This hypofractionation approach yields a dose equivalent at 2 Gy fractionation (EQD_2Gy_) of 43.6 Gy total dose to the breast and an EQD_2Gy_ of 56.7 Gy to the tumor bed assuming an α/β ratio of 3.5 Gy for breast cancer [2].

Radiotherapy could be given either as 3D conformal RT (3D-CRT) or as intensity-modulated radiotherapy (IMRT), including rotational techniques such as volumetric modulated arc therapy (VMAT). Radiotherapy was delivered by a linear accelerator with a minimal energy of 6 MeV using either photon/electron or photon/photon combinations depending on optimal PTV coverage. Dose constraints were median dose to the ipsilateral lung <10 Gy, median dose to the heart <5 Gy, median and maximum to the left anterior descending coronary artery (RIVA) <15 and ≤40 Gy, and median dose to the contralateral breast <3 Gy. Deep-inspiration breath-hold techniques or other heart-sparing techniques for left-sided cancers were allowed but not specifically recommended [18, 19].

### Toxicity assessment

Toxicity was evaluated by the treating physicians at baseline prior to radiotherapy, on the first treatment day, and thereafter in weekly intervals during radiotherapy as well as 4 to 6 weeks and 6 months after radiotherapy using the National Cancer Institute Common Toxicity Criteria (NCI-CTC), version 4.03 [20]. In addition, the item “feeling of pressure” (i.e., in the breast) was studied, which is not a predefined CTCAE item. However, our previous work identified this as a relevant toxicity occurring in approximately 10% of patients [13]. Grading was performed in analogy with CTCAE criteria, version 4.03 [20]. Quality of life was assessed by EORTC QLQ-C30 [21] and EORTC QLQ-BR23 [22] questionnaires at the same timepoints, results will be reported elsewhere. Performance status was measured using the ECOG scale until the end of treatment. Cosmesis was rated by physicians and patients separately at the start and end of radiotherapy and at 6 months using the four-tiered Radiation Therapy Oncology Group/Harvard Scale [23].

### Study design and statistical assumptions

The primary endpoint was acute toxicity. The study hypothesis was that the frequency of acute skin reactions grade ≥2 at any timepoint would be 20% or less. Under the assumptions of a two-sided significance level of 5% using the chi-squared test, 80% power, observed proportion 11.1%, and screening failure rate of 7.3%, a sample size of 150 patients was calculated. The study aimed to recruit these 150 patients within a recruitment period of 1 year.

Missing toxicity and safety data were imputed by the last observation reported (last observation carried forward method). Missing data on feasibility were counted as not feasible. Modified score function 95% confidence intervals were computed. Descriptive statistics are mean ± standard deviation (SD) for nearly normal distributions or median with interquartile range.

### Administrative aspects

The study was approved by the ethics committee of the University of Lübeck (leading ethics committee) as well as by the ethics committees which were responsible for the participating sites. The trial was registered in a clinical trial database (www.clinicaltrials.gov) under the registration number NCT01948726 and was supported the by the ARO (*Arbeitsgemeinschaft Radiologische Onkologie*, German Radiation Oncology clinical research group).

## Results

**Fig. 1:**
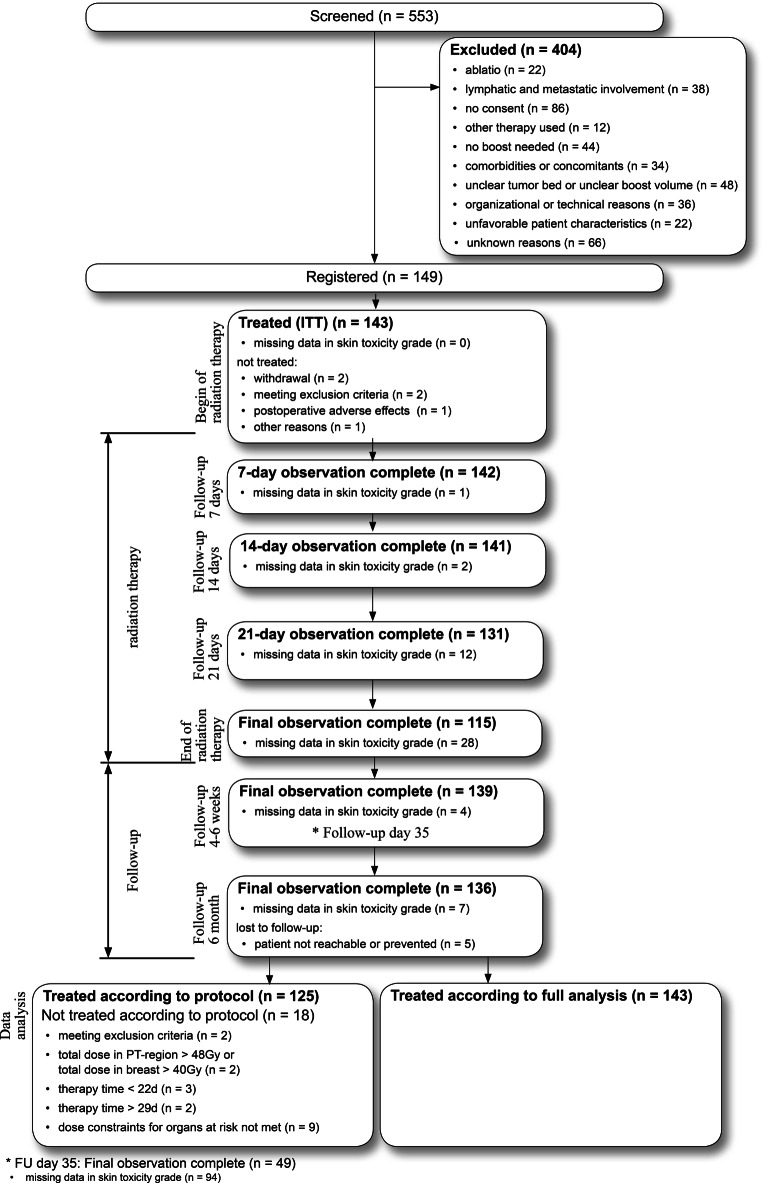
Flow diagram for the trial. *ITT* Intention to treat, *PT* primary tumor

From November 2013 to July 2014, 149 patients were recruited at 12 participating centers (one academic institution, six non-academic hospital-based radiation oncology departments, five private practices). Two patients withdrew consent after inclusion, two patients were excluded because of new findings during treatment planning with subsequent changes in therapy, and one patient each was not treated because of a postoperative adverse event occurring before initiation of radiotherapy and for an unknown reason (Fig. 1, flowchart), leaving 143 patients for analysis. The number of patients per site ranged from 1 to 38. Recruitment was much faster than expected and completed within 8 months (expected: 12 months). Patients’ characteristics are listed in Table 1.

**Table 1:** Baseline characteristics of the 143 treated patients

*Age*	62.0 ± 9.9 years
	Median: 61 years
	Range: 40–85 years
*Body mass index*	26.5 ± 4.5 kg/m^2^
	Median: 26 kg/m^2^
	Range: 16–43.9 kg/m^2^
*ECOG-PS*	
0	134 (93.7%)
1	9 (6.3%)
*Type of breast surgery*	
Tumorectomy	118 (82.5%)
Tumorectomy plus oncoplastic surgery	24 (16.8%)
Other	1 (0.7%)
*Frequency of re-resection*	
0	107 (74.8%)
1	17 (11.9%)
2	9 (6.3%)
More than 2 (maximum 5)	9 (6.3%)
Unknown	1 (0.7%)
*Minimal resection margin*	Mean: 4 mm
	Range: 0–13 mm
*Axillary surgery*	
Sentinel lymph node biopsy	136 (95.1%)
Axillary dissection	7 (4.9%)
*Number of removed lymph nodes*	Median: 2
	Range: 0–23
*Tumor size*	Median: 14 mm
	Range: 2–53 mm
*T‑category*	
ypT0	2 (1%)
pT1a/b	45 (32%)
pT1c	69 (48%)
pT2	27 (19%)
*N‑category*	
pN0	136 (95%)
pN1a	7 (5%)
*Histology*	
Invasive ductal	111 (79%)
Invasive lobular	22 (16%)
Medullary	2 (1%)
Others	6 (4%)
Unknown	2 (1%)
*Extensive intraductal component*	
Yes (present)	4 (3%)
No	83 (58%)
Unknown/no data	56 (39%)
*ER-status*	
Positive	124 (87%)
Negative	15 (10%)
Missing	4 (3%)
*HER‑2 status*	
0	71 (50%)
1+	49 (34%)
2+	15 (10%)
3+	8 (6%)
*Chemotherapy*	
None	94 (66%)
Adjuvant	38 (27%)
Neoadjuvant	8 (6%)
Unknown	3 (2%)
*Adjuvant endocrine therapy*	
Yes, during radiotherapy	41 (29%)
Yes, scheduled after radiotherapy	79 (55%)
No	18 (13%)
Unknown	5 (3%)
*Type of endocrine therapy*	
Tamoxifen	54 (38%)
Aromatase inhibitors	44 (31%)
Unknown	45 (32%)

The prescribed number of 16 radiotherapy fractions was applied in all patients. The administered mean dose to the breast PTV was 40.01 ± 0.12 Gy and the mean dose to the boost volume was 48.01 ± 0.08 Gy. The mean overall treatment time for all patients was 23.1 ± 2 days (median 22 days, range 21–32 days, more than 29 days in 2 patients). Thus, the adherence to protocol-specified dose prescription and treatment time was high. Compliance with dose constraints was 100% regarding median dose to the ipsilateral lung <10 Gy; 100% for median heart dose <5 Gy; 94% for median and 99% for maximum dose to the RIVA <15 and ≤40 Gy, respectively; and 100% for median dose to the contralateral breast <3 Gy. Eighty-four patients (58.7%) were treated with 3D-CRT whereas 59 (41.3%) were treated with IMRT.

Acute skin toxicity (the primary endpoint) was evaluated in 143 patients. 122 patients never experienced skin toxicity greater than grade 1. In 21 patients (14.7%, 95% confidence interval 9.8–21.4%) the highest reported grade of skin toxicity at any visit during the whole study period was either grade 2 (*n* = 19) or grade 3 (*n* = 2). There was no grade 4 skin toxicity. Since the upper border of the 95% confidence interval was greater than 20%, the primary hypothesis could not be confirmed at the predefined significance level of 5%.

**Fig. 2:**
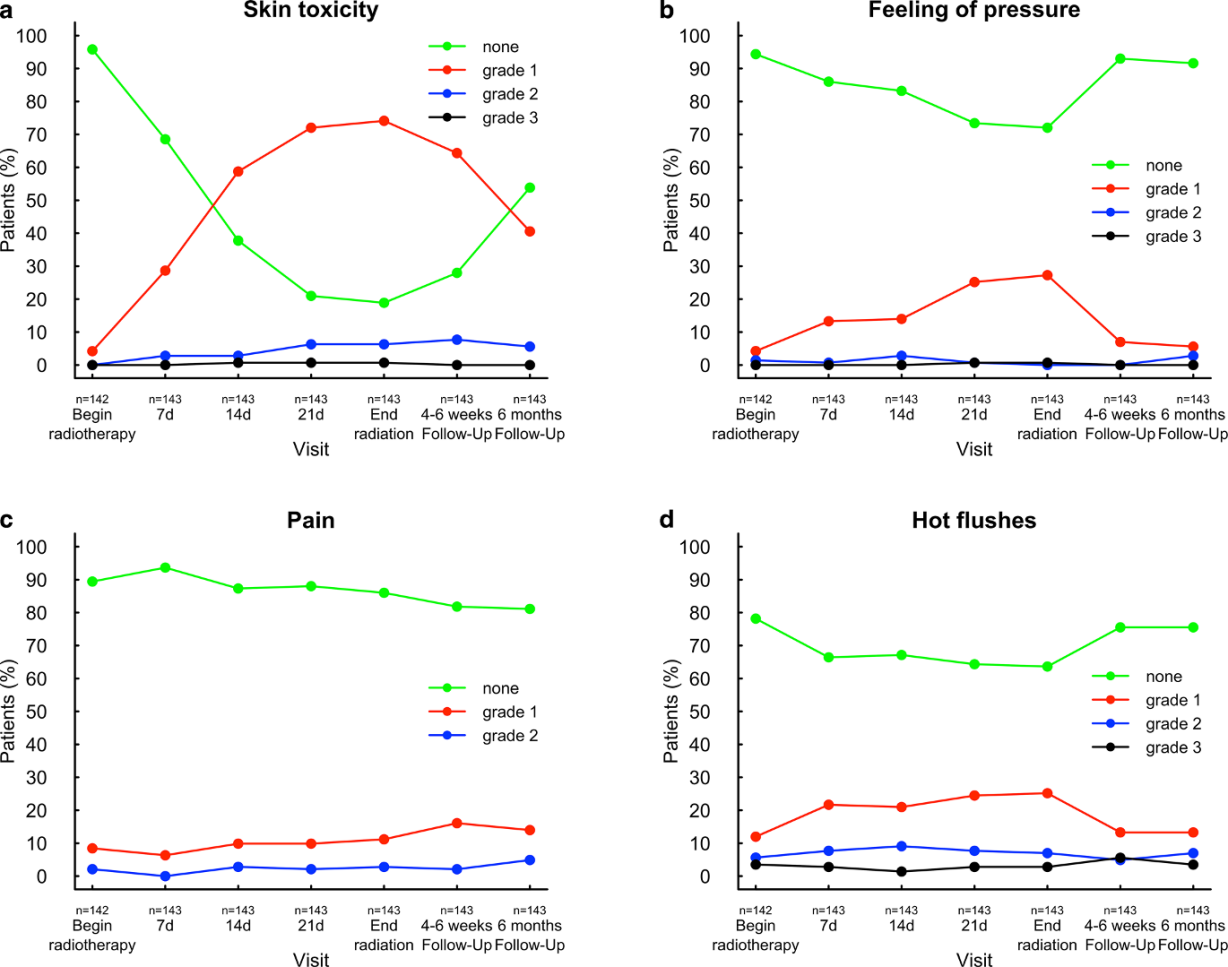
Course of toxicity for skin toxicity (**a**), feeling of pressure (**b**), breast pain (**c**), and hot flashes (**d**) by toxicity grade and study visit

Other acute toxicities are listed in Table 2. The most frequent grade 3 toxicity (11%) were hot flashes related to endocrine therapy. In 4% of patients, these were present prior to radiotherapy. There was no grade 4 toxicity. Fig. 2 depicts the course of skin toxicity, feeling of pressure, breast pain, and hot flushes by toxicity grade and study visit. ECOG performance status was constant, with 94% of patients having an ECOG of 0. Only 1 patient had a decline from ECOG 0 to ECOG 1 at the end of treatment.

**Table 2:** Maximum toxicity grades (NCI-CTC score) during radiotherapy and in the first 6 weeks after radiotherapy

Toxicity	*N* (%)	Grade 0 (%)	Grade 1 (%)	Grade 2 (%)	Grade 3 (%)	Grade 4 (%)	Missing
Skin reaction	143	9	76	13	1	0	0
Nausea	142	84	13	2	1	0	1
Pain	143	64	29	8	0	0	0
Hot flashes	142	54	26	8	11	0	1
Swelling, pressure	142	60	36	4	1	0	1

The only serious adverse event recorded was a fracture of the upper arm that was not related to the study treatment. After open repositioning of multiple fragments, the patient fully recovered. Only one severe adverse event was classified as treatment related (skin reaction grade three), but fully recovered with no residual signs at next follow-up.

Cosmesis prior to radiotherapy was estimated by the physician/patient as excellent in 40%/34%, good in 51%/55%, fair in 6%/5%, and poor in 1%/1% (missing data in 2%/5%). Cosmesis was re-evaluated by the end of radiotherapy as well as 6 weeks and 6 months after the end of radiotherapy. There were no changes in cosmetic outcome over time. Six months after radiotherapy, cosmesis was classified by the physician/patient as excellent in 40%/34%, good in 51%/57%, fair in 6%/5%, and poor in 1%/1% (missing data in 2/3%, respectively).

## Discussion

Our results show that hypofractionated whole-breast radiotherapy with SIB is safe and feasible with low rates of acute toxicity.

The SIB has some dosimetric advantages [6, 24, 25]. Dose homogeneity within the breast tissue outside the boost volume is increased. So far, data from several prospective trials using SIB in combination with conventional fractionation to the breast have been published. The largest experience comes from the University of Groningen [8, 26]. A simultaneous integrated boost (SIB) was used in 982 patients. Local control was exceptionally good, with a 5-year local recurrence rate of 1.8%. After 36 months, 8.5 and 3.7% of patients had grade ≥2 fibrosis in the boost area and grade ≥2 telangiectasia, respectively.

The use of hypofractionated whole-breast radiotherapy with SIB is considered investigational at the moment. Data from small prospective series have been reported [11–14].

A randomized controlled phase II trial conducted at the University of Brussels [27] enrolled 69 patients and randomized them to conventionally fractionated tangential whole-breast radiotherapy with a sequential boost (25 × 2 Gy plus 8 × 2 Gy) or hypofractionated tomotherapy with a SIB (15 × 2.8 Gy to the whole breast + additional 0.6 Gy SIB). Acute radiation dermatitis was similar between the two arms; however, patients treated in the control arm had a trend towards more skin changes 2 years after treatment (60% vs. 30%; *p* = 0.06). At 2 years, changes in lung diffusion capacity were significantly more frequent in the experimental arm (29.2% vs. 7.4%; *p* = 0.047).

In another randomized controlled trial [15], 167 patients were randomized to prone hypofractionated whole-breast radiotherapy with 40.05 Gy in 15 fractions with a sequential boost (10 Gy in 4 fractions with clear margin or 14.88 Gy in 6 fractions in case of positive margins) or a SIB (additional daily dose of 0.45 Gy or 0.66 Gy). Acute radiation dermatitis grade ≥2 (45.8% vs. 28.9%; *p* = 0.037) and pruritus (61% vs. 43%; *p* = 0.015) occurred significantly more often in patients in the control arm; however, there was no difference regarding moist desquamation, the primary endpoint of the trial.

Larger randomized studies are on the way, but results are pending. RTOG 1005 has already completed recruitment and results are expected in the coming years. Another approach to shorten overall treatment time is application of an intraoperative tumor bed boost. A combination of hypofractionated whole-breast radiotherapy and intraoperative electron radiotherapy was studied in the HIOB-trial (NCT01343459). First results were recently published and showed favorable local control and low rates of toxicity [28].

Our study was performed as a pilot study for preparation of a larger randomized controlled phase III trial (HYPOSIB; NCT02474641) with the objective of evaluating the acute toxicity of hypofractionation plus SIB in a multicenter setting. Protocol adherence was very high, with no major deviations. Acute skin reaction grade ≥2 occurred in 14.7% (95% confidence interval 9.8 to 21.4%) of patients. Nevertheless, the primary hypothesis could not be confirmed at the 5% significance level. Overall toxicity was low. Only one severe adverse event was reported, and this was not related to radiotherapy. Acute grade 3 toxicity was, in the majority of cases, related to concomitant adjuvant endocrine therapy. However, follow-up is still short and no conclusions on local recurrence rates and long-term toxicity can be drawn from the presented data [29, 30].

In summary, this study demonstrated that hypofractionated adjuvant radiotherapy with SIB for patients with breast cancer is feasible and safe in terms of acute toxicity. A randomized controlled phase III trial (HYPOSIB; NCT02474641) is ongoing and has completed accrual of 2324 patients.
